# Functional Interaction between CFTR and the Sodium-Phosphate Co-Transport Type 2a in *Xenopus laevis* Oocytes

**DOI:** 10.1371/journal.pone.0034879

**Published:** 2012-04-13

**Authors:** Naziha Bakouh, Baya Chérif-Zahar, Philippe Hulin, Dominique Prié, Gérard Friedlander, Aleksander Edelman, Gabrielle Planelles

**Affiliations:** 1 Inserm UMRS-845, Paris, France; 2 Université Paris Descartes, Sorbonne Paris Cité, Faculté de Médecine Paris Descartes, Paris, France; University of Chicago, United States of America

## Abstract

**Background:**

A growing number of proteins, including ion transporters, have been shown to interact with Cystic Fibrosis Transmembrane conductance Regulator (CFTR). CFTR is an epithelial chloride channel that is involved in Cystic Fibrosis (CF) when mutated; thus a better knowledge of its functional interactome may help to understand the pathophysiology of this complex disease. In the present study, we investigated if CFTR and the sodium-phosphate co-transporter type 2a (NPT2a) functionally interact after heterologous expression of both proteins in *Xenopus laevis* oocytes.

**Methodology/Findings:**

NPT2a was expressed alone or in combination with CFTR in *X. laevis* oocytes. Using the two-electrode voltage-clamp technique, the inorganic phosphate-induced current (IPi) was measured and taken as an index of NPT2a activity. The maximal IPi for NPT2a substrates was reduced when CFTR was co-expressed with NPT2a, suggesting a decrease in its expression at the oolemna. This was consistent with Western blot analysis showing reduced NPT2a plasma membrane expression in oocytes co-expressing both proteins, whereas NPT2a protein level in total cell lysate was the same in NPT2a- and NPT2a+CFTR-oocytes. In NPT2a+CFTR- but not in NPT2a-oocytes, IPi and NPT2a surface expression were increased upon PKA stimulation, whereas stimulation of Exchange Protein directly Activated by cAMP (EPAC) had no effect. When NPT2a-oocytes were injected with NEG2, a short amino-acid sequence from the CFTR regulatory domain that regulates PKA-dependent CFTR trafficking to the plasma membrane, IPi values and NPT2a membrane expression were diminished, and could be enhanced by PKA stimulation, thereby mimicking the effects of CFTR co-expression.

**Conclusion/Perspectives:**

We conclude that when both CFTR and NPT2a are expressed in *X. laevis* oocytes, CFTR confers to NPT2a a cAMPi-dependent trafficking to the membrane. This functional interaction raises the hypothesis that CFTR may play a role in phosphate homeostasis.

## Introduction

The Cystic Fibrosis Transmembrane conductance Regulator, CFTR (ABCC7, encoded by *CFTR* gene), is a cAMP-stimulated channel that mediates the transmembrane transport of chloride in epithelial cells, thereby participating in transepithelial transport. The importance of CFTR in cell and organ physiology has been proven by the deleterious consequences of CFTR mutations that lead to Cystic Fibrosis (CF), an autosomal genetic disease. CF phenotype is dominated by alterations in epithelial secretions. These abnormal secretions are related to CFTR defects, in a direct (defect in CFTR function) or indirect manner (loss of functional interactions between CFTR and ion transporters). The loss of interactions between CFTR and other ion transporters have important consequences: the poor hydration of airways mucus and the reduced alkalization of pancreatic juice during CF are related to the loss of interaction between CFTR and the epithelial Na^+^ channel (ENaC) or between CFTR and the Cl^-^/HCO_3_^-^ exchangers (SLC26A3 and A6), respectively [1], [2]. Other dysfunctions may be more subtle. For example, it had been long thought that despite the wide expression of CFTR along the human nephron, there was no detectable CF renal phenotype [3], [4]. But later it was shown that the loss of interaction of CFTR with megalin could lead to a defective receptor-mediated endocytosis in the renal proximal tubule, thus an enhanced urinary transferrin loss during CF [4]. In this nephron segment, CFTR is colocalized with the sodium-phosphate co-transporter NPT2a (encoded by *SLC34A1* gene), as it is in osteoblasts [4], [5], [6]. By mediating the coupled influx of 3 Na^+^ and 1 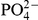 into the cell, NPT2a is responsible for a large part of the adult renal phosphate absorption and participates in bone mineralization. Interestingly, early-onset reduction in bone mineral density is observed during CF, but the direct involvement of CFTR in mineral balance has been difficult to demonstrate due to the marked nutritional problems of the patients [7]. Also, microscopic nephrocalcinosis and higher incidence of renal stones have been reported in CF [8], [9]. Concurrent factors such as hypocitraturia or aminoglycoside treatment [4] make it difficult to delineate the precise role of CFTR in these disorders. Nonetheless, these observations raise the question of a possible interaction between CFTR and NPT2a.

To investigate whether CFTR expression interacts with NPT2a function, we co-expressed both proteins in *Xenopus laevis* oocytes. Using the two-electrode voltage-clamp technique, the current induced by inorganic phosphate (IPi) was measured and taken as an index of NPT2a-mediated Pi transport [10]. In oocytes expressing both CFTR and NPT2a (NPT2a+CFTR-oocytes), IPi was significantly lower than in oocytes expressing NPT2a alone (NPT2a-oocytes). Experimental conditions that stimulated PKA induced a rise in IPi in NPT2a+CFTR-oocytes, but not in oocytes expressing NPT2a alone. The PKA-induced increase in IPi was associated with an increase in the membrane expression of NPT2a in NPT2a+CFTR-oocytes. Interestingly, CFTR and NPT2a were found to co-immunoprecipitate, suggesting that both proteins lie in close proximity in a cell compartment. This prompted us to check the effect of injecting NEG2 in NPT2a-oocytes: this short peptide from the CFTR regulatory (R) domain (residues 817–838) was shown to regulate the cAMPi-induced CFTR trafficking from a regulated intracellular compartment to the plasma membrane [11]. In NPT2a-oocytes, the injection of NEG2 mimicked the effects of CFTR co-expression: basal IPi was reduced, and was stimulated by cAMPi increase. These results suggest that, after expression in *X. laevis* oocytes, CFTR expression functionally affects the function of NPT2a by inducing a PKA-dependent traffic of NPT2a to the oocyte plasma membrane.

## Methods

### Biological Material

Maintenance and experiments on female *Xenopus laevis* were carried out in strict accordance with the French laws on Laboratory Animals. The protocol was approved by the Ethical Committee for Animal Experiments of Ile-de-France Paris-Descartes University (Permit Number: P2.GP.088.09). All efforts were made to minimize suffering. Anesthesia was achieved by the brief immersion of *X. laevis* in iced water supplemented with 2 mM ethyl-*n*-aminobenzoate-methane sulfonate and was maintained by cooling the toad on ice during the partial ovariectomy. Oocyte defolliculation was achieved by the gentle shaking (2 h, RT) of small ovarian fragments in calcium-free ND96 (ND 96 composition in mmol/l: 96 NaCl, 2 KCl, 1 MgCl_2_, 1 CaCl_2_, 5 Hepes, adjusted to pH 7.5 with NaOH) and supplemented with 0.4 U/ml collagenase (1A, Sigma). Selected stage V-VI oocytes were injected (Inject+Matic microinjector, Geneva, Switzerland) with RNAs dissolved in RNAse-free water (50 nl), or with water alone (H_2_O-oocytes), and incubated at 18°C in ND96 supplemented with penicillin/streptomycin for 3–4 days before experiments.

### Heterologous Expression of CFTR and NPT2a (Plasmid constructs and cRNA preparation)

Human NPT2a cDNA was cloned into the *Xenopus* expression vector pSP64T. In the Myc-NPT2a construct, we added an in-frame c-Myc sequence at the NPT2a 5′ end. Human CFTR cDNA was cloned into pT7TS. Capped RNAs were synthesized *in vitro* from the linearized constructs using mMESSAGE mMACHINE® (Ambion, Austin, TX, USA). Except when indicated, oocytes were injected with 10 ng of NPT2a cRNAs [10] and/or with 1 ng of CFTR cRNAs [11].

### Two-electrode Voltage-clamp Experiments (TEVC)

Once placed in a microchamber, oocytes were punctured with two low-resistance (0.5–1 Mohm), 3M KCl-filled microelectrodes. A two-bath electrode configuration was used to reduce series resistance-induced errors during voltage-clamp measurements: a virtual ground amplifier (VG2-A 100, Axon Inst, Union City, CA, USA) was connected to the current-voltage amplifier (Axoclamp 2B, Union City, CA, USA) and to the bath electrodes (an agar-3M KCl bridge electrode and an Ag-AgCl pellet) [12],[13]. Whole cell currents were recorded on a multichart recorder (Arc en Ciel, Sefram, Servofram, France) by holding the membrane potential value at Vc  =  −50 mV. Current voltage (I/V) relationships were obtained by applying voltage steps ± 20 mV (5 seconds duration, range –100 to +60 mV) from the resting membrane potential (V_m_), using Clampex9-generated protocol. Results were interfaced with Digidata 1322A to a computer, and were analyzed with the P-Clamp9 software program (Axon Instruments, USA). Whole cell membrane conductance, G_m_, was calculated from I/V curves.

### Solutions and Reagents

Oocytes were basally superfused with ND96. Solution change was commanded electronically, using a laboratory-made device. Unless otherwise noted, IPi was induced by the reversible addition of 1 mM Pi in ND96 (pH 7.5). PKA was stimulated by using a mixture of ND 96 supplemented with 1 µM forskolin (Forsk) and 100 µM isobutylmethylxanthine (IBMX); H89 was used to inhibit PKA. Preliminary experiments showed that using the inactive derivative 1,9 dideoxyforskolin in place of Forsk had no effect, consistent with a specific effect of the active form. The increase in intracellular adenosine 3′, 5′ cyclic monophosphate (cAMP_i_) was also provoked using the membrane permeant analogs 8 Bromoadenosine-3′5′-cyclic monophosphate (8-Br-cAMP), N^6^-monobutyryladenosine-3′, 5′-cyclic monophosphate (6-MB-cAMP), para-Chlorophenylthio-2′-O-methyladenosine-3′, 5′-cyclic monophosphate (8-pCPT-2′-O-Me-cAMP), and N^6^-Mono-t.butylcarbamoyladenosine-3′, 5′-monophosphate (6-MBC-cAMP). To obtain the synergistic activation of A and B sites of PKA I and II isozymes, the membrane permeant 8-hexylaminoadenosine 3′, 5′-cyclic monophosphate (8-HA-cAMP), and the Sp isomer 8-piperidinoadenosine-3′, 5′-cyclic monophosphorothioate (Sp-8-PIP-cAMP) were combined in a 10∶1 ratio with 6-MB-cAMP and 6-MBC-cAMP, respectively. All c-AMP derivatives were purchased from Biolog Life Sci Inst (Bremen, Germany). PKC stimulation was obtained by supplementing ND96 with 50 nM Phorbol 12-Myristate 13-Acetate (PMA). The selective CFTR blocker CFTR-Inh*172 [14] was obtained from Calbiochem. Other products were from Sigma. When necessary, drugs were dissolved in DMSO or ethanol (final concentration <0.1% vol/vol; vehicule alone was added in ND96). The exposure to a drug was mostly achieved by continuous superfusion. However, when several minutes of exposure was planned, incubation was preferred to free flow superfusion due to the cost. In such cases, the drug was directly added into the microchamber of the electrophysiological experiment; to this end, the flow of the superfusion and the synchronous aspiration were suspended, then re-initiated to perform the electrophysiological measurement as described elsewhere [15].

Human NEG2 peptide (GLEISEEINEEDLKECFFDDME) or a scrambled sequence, sNEG, (LIKEFSEEDGECLMIDEDENEF) were synthesized by Proteogenix (Oberhausbergen, France), and dissolved at the 12.5 mM concentration in an intracellular-like medium (in mM, Na Glutamate 128, NaCl 5, MgSO_4_ 7, 20 Hepes/KOH pH 7.0), and stored at −20°C [11]. On the day of the experiment, 50 nl of the peptide-containing solution (NEG2 or sNEG2) was injected into oocytes as detailed in [11]. IPi was measured 2 hours after the peptide injection.

### Expression Analysis

To analyze the expression level of NPT2a, oocytes were injected with 10 ng of Myc-NPT2a cRNA (preliminary experiments showed that the Myc tag on the N terminal region of NPT2a did not alter the functional properties of NPT2a). Western blot analysis on total proteins and on biotinylated plasma membrane proteins was adapted from [16], [17]. 30–40 oocytes were washed in iced PBS, then biotinylated by a 30 min gentle rotation in cold 0. 1 mM CaCl_2_- and 1 mM MgCl_2_-containing PBS supplemented with 1 mg/ml sulfo-NHS-SS-Biotin (Pierce Biotechnology, Rockford, IL, USA), pH 9.2. A 4-times washing in a biotin-quenching solution (glycine 192 mM, Tris HCl 25 mM, pH 7.5 in PBS) was followed by lysis of the oocytes in 250 mM sucrose, 0.5 mM EDTA, 5 mM Tris-HCl (pH 7.4), supplemented with a protease inhibitor cocktail (Complete Mini, Roche, Indianapolis, IN, USA). Cell lysates were centrifuged at 200, 400 and 800 g (10 min each) to eliminate the yolk, and a sample of the resulting supernatant (corresponding to 2 oocytes) was diluted in SDS-Laemmli buffer for the immunoblotting of total proteins. The remainder was centrifuged at 14 000 g to isolate total membranes. The supernatant was incubated overnight with Monomeric Avidin beads (Pierce Biotechnology, Rockford, IL, USA) pre-washed in lysis buffer. Beads were washed (4 times) with the lysis buffer, suspended in 50 µL 2X Laemmli, and heated for 5 min (90°C) before being loaded. Samples of proteins were separated by SDS-8% polyacrylamide gel electrophoresis, and transferred to nitrocellulose membranes (BioRad, Marnes la Coquette, France). Non-specific binding was prevented by incubating (1 h, at room temprature, RT) the membranes with 1% low fat milk and 1% bovine serum albumin in PBS, supplemented with 0.5% Tween-20 (PBST). Membranes were probed overnight (4°C) with a primary anti-Myc antibody (Ab) (Invitrogen, Carlsbad, IL, USA), diluted to 1∶5000. The same procedure was used to analyze the level of CFTR plasma membrane expression in oocytes injected with Myc-NPT2a and CFTR RNAs, using an anti-CFTR Ab (anti-CFTR clone MM13-4 monoclonal antibody, Millipore, Temecula, CA, USA) diluted to 1∶1000. Sheep anti-mouse IgG, HRP-conjugated secondary Ab diluted to 1∶5000 (Amersham Biosciences, Buckinghamshire, UK) was applied for 1 hour to the membranes (RT). Protein loading control was checked on the same membrane using a mouse anti-actin Ab (Santa Cruz Biotechnology Inc, Santa Cruz, CA, USA) diluted to 1∶1000. Stained proteins were detected using an enhanced chemoluminescence system (GE Biotechnologies, Orsay, France). Films were recorded digitally and quantified using the NIH image J 1.42 q software (National Institutes of Health, Bethesda, MD, USA) (available at http://rsb.info.nih.gov/).

### Immunoprecipitation and Co-immunoprecipitation Assays

The protocol used for immuno- and co-immunoprecipitation was adapted from [18], [19]. Briefly, 3 days after being injected with 10 ng cRNA coding for Myc-NPT2a and for CFTR, 80 oocytes were lysed and centrifuged at 200, 400 and 800 g as described above. The final supernatant was centrifuged at 10 000 g for 30 min. Immunoprecipitation of the antigens was achieved by a 60 min rotating incubation (RT) of the resulting supernatant with 2 µg of anti-Myc or anti-CFTR clone MM13-4 Ab, or with IgG1 Ab as a negative control (Millipore, Temecula, CA, USA), followed by the addition of G Protein-conjugated magnetic beads, 20 µl (Bio-Adembead Protein G, Ademtech, Pessac, France) for 30 min. The magnetic beads were suspended in 20 µl of lysis buffer and washed 3 times before elution in Laemmli sample. Proteins were resolved by SDS-PAGE and transferred to nitrocellulose membranes for Western blot analysis. Anti-Myc Ab diluted to 1∶5000 (when antigens were immunoprecipitated using anti-CFTR Ab) or, conversely, anti-CFTR diluted to 1∶1000 (when antigens were immunoprecipitated using anti-Myc Ab) were applied (1 hour, RT). Proteins were detected using sheep anti-mouse IgG diluted to 1∶5000 coupled to horseradishperoxidase HRP.

### Statistics

Except when stated, results were expressed as means ± SEM, with n as the number of oocytes, and N the number of experiments (from different donors). To eliminate possible experimental variations related to the different donors, IPi was normalized to IPi value measured in basal conditions in NPT2a-oocytes from the same batch. Significance of the results was assessed by paired or unpaired Student t-test using SigmaPlot (Systat software Inc., San Jose, CA). The difference was considered significant for a P value <0.05.

## Results

### Functional Expression of NPT2a and CFTR in X. Laevis Oocytes

The successful functional expression of CFTR or of NPT2a was assessed in oocytes expressing each protein alone, or in combination. To this end, whole cell current was continuously monitored at Vc =  −50 mV, or by applying 20 mV voltage steps from the resting V_m_. Exposure of CFTR- or NPT2a+CFTR-oocytes to a Forsk+IBMX mixture or to 100 µM 8-Br-cAMP induced within 15 min a large G_m_ increase: at Vc =  –50 mV, G_m_ increased from 1.36 ± 0.77 to 9.06 ±3.12 µS, n  = 10 (Forsk+IBMX condition) and from 1.61±0.28 to 10.75±2.93 µS (8-Br-cAMP condition). The induced G_m_ increase was inhibited by using a CFTR inhibitor, CFTR-Inh*172, 20 µM as shown in **Figure S1**. Forsk+IBMX or 8-Br-cAMP were without effect in NPT2a- or in H_2_O-oocytes (Vc =  -50 mV, n =  10 for each type of oocytes). These results are consistent with the activation in CFTR-and NPT2a+CFTR-oocytes of a CFTR-mediated current, ICFTR, triggered by a cAMPi increase.

**Figure 1:**
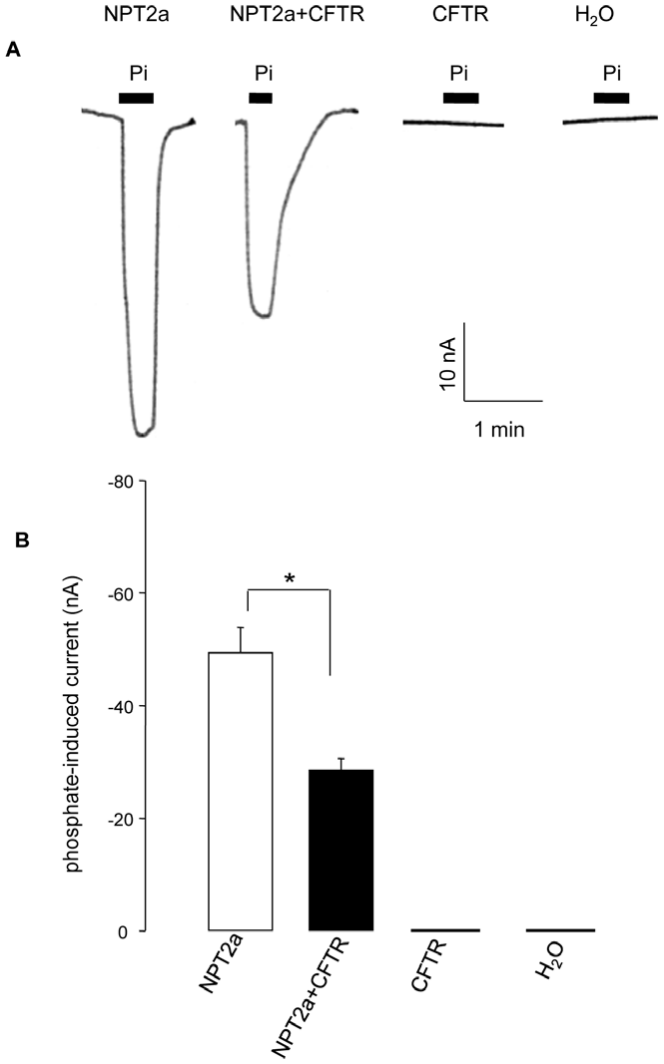
Phosphate-induced current in *X. laevis* oocytes. Phosphate-induced current (1 mM of Pi added in ND96) was measured at holding potential (Vc) of −50 mV in oocytes expressing NPT2a or CFTR alone or co-expressing NPT2a and CFTR; oocytes injected with water were used as control oocytes. A: Original tracings showing the current induced by the addition of 1 mM Pi (indicated by black bars) in the superfusing medium. The type of oocytes is indicated above the tracings. B: Summary of the results (means ± SEM) of the effect of 1 mM Pi in the different types of oocytes (indicated below the columns). Significance of the results *: P<0.05

Addition of 1 mM Pi in ND96, at Vc = −50 mV, induced an inward current in both NPT2a- and NPT2a+CFTR-oocytes and was without effect in CFTR-oocytes (n  =  15) or in H_2_O-oocytes (n  =  20). **Figure 1** shows original tracings from this experimental series (**Figure 1A**). IPi was significantly larger in oocytes expressing NPT2a alone than in oocytes co-expressing NPT2a and CFTR (−49.4±4.5 nA, n = 15 vs −28.6±2.1 nA, n  =  14, **Figure 1B**). This difference was further investigated.

IPi was measured in NPT2a- and in NPT2a+CFTR-oocytes from a same batch of oocytes from the 2^nd^ day to the 7^th^ day after cRNAs injection. For NPT2a-oocytes, IPi was -62.7±2.8 nA (n = 5) at day 2, not different from −59.5±6.9 nA (n =  7) at day 7; for NPT2a+CFTR-oocytes, IPi was −42.8±9.2 nA (n =  5) at day 2, not different from –44.5±6.4 nA (n =  5) at day 7. These results indicate that CFTR co-expression did not delay the full expression of NPT2a. In a separate series, oocytes were injected with 1 ng CFTR cRNA and with increasing amounts of cRNA coding for NPT2a, i.e., 10 ng (n  =  9), 20 ng (n  =  6) or 30 ng (n  =  8), and IPi was measured. IPi rose by 31.9±2.4% from 10 to 20 ng RNA, and by 49.0±4.0% from 10 to 30 ng RNA, in good agreement with previous results from our lab on the effect of increasing cRNA in oocytes expressing NPT2a alone (10). These results ruled out the possibility that failure of oocyte machinery to co-express proteins was responsible for a sub optimal expression of NPT2a, thus the reduced IPi. Further investigations were performed by injecting 10 ng NPT2a cRNAs and 1 ng CFTR cRNA.

**Figure 2:**
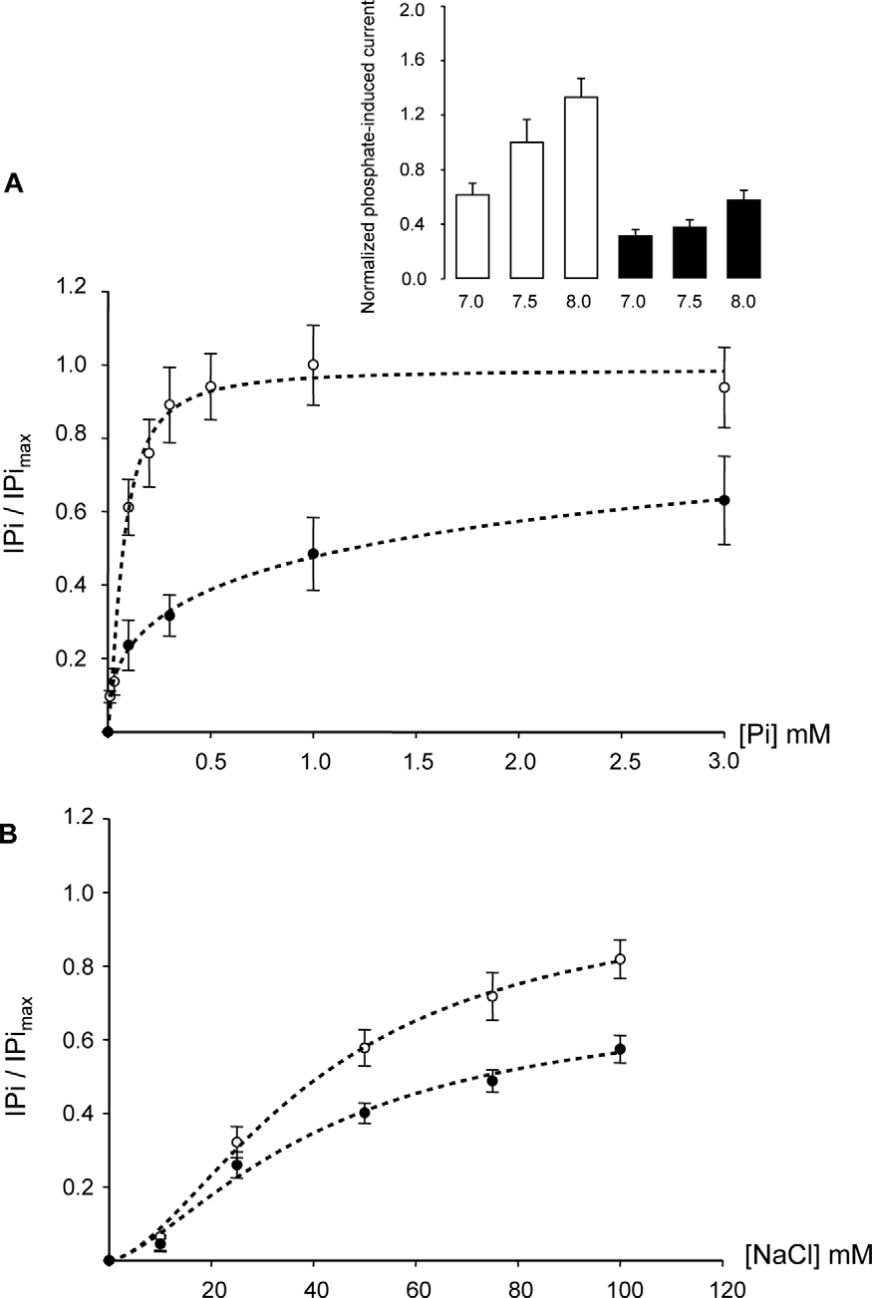
Kinetic analysis of inorganic phosphate transport in oocytes expressing NPT2a or co-expressing NPT2a and CFTR. Phosphate-induced currents (IPi) were measured (Vc =  −50 mV) in NPT2a-oocytes (empty circles) and in NPT2a+CFTR-oocytes (filled circles). The dashed lines through the points (means ± SEM from n  =  6 to 14, N  =  5) were calculated using SigmaPlot software (Systat software Inc., San Jose, CA). A: IPi was induced by increasing Pi concentration in ND 96, pH 7.5, as indicated in abscissa and normalized against extrapolated maximal current (IPi_max_). Data were fitted to Michaelis-Menten equation. Results, as means ± SD, were as follows: the apparent concentration of Pi substrate (K_m_) giving the half IPi_max_ was not changed by CFTR expression (K_m_  =  0.07± 0.02 *vs* 0.06 ± 0.02 mM in NPT2a- and NPT2a+CFTR-oocytes, respectively), but IPi_max_ was decreased by 40±3% (P <0.05). B: IPi was induced by 1 mM Pi at increasing Na^+^ concentrations (equimolary substituted by choline^+^, pH 7.5) and normalized against IPi_max_. Data were fitted to the modified Hill equation. Results, as means ± SD, were as follows: the apparent K_m_ for Na^+^ substrate was 40.7 ± 14.6 in NPT2a-oocytes, not different from 41.8 ± 13.4 mM in NPT2a+CFTR-oocytes; IPi_max_ was decreased by 39±7% in NPT2a+CFTR-oocytes compared to NPT2a-oocytes (P < 0.05). Inset: Effect of varying extracellular pH from 7.0 to 8.0 on the current induced by 1 mM Pi in NPT2a-oocytes (white column) and in NPT2a+CFTR-oocytes (black column). IPi was normalized against the Pi-induced current measured at pH 7.5 in NPT2a-oocytes from the same batch of oocytes. Results are shown as means± SEM, n  =  5 oocytes of each type.

**Figure 3:**
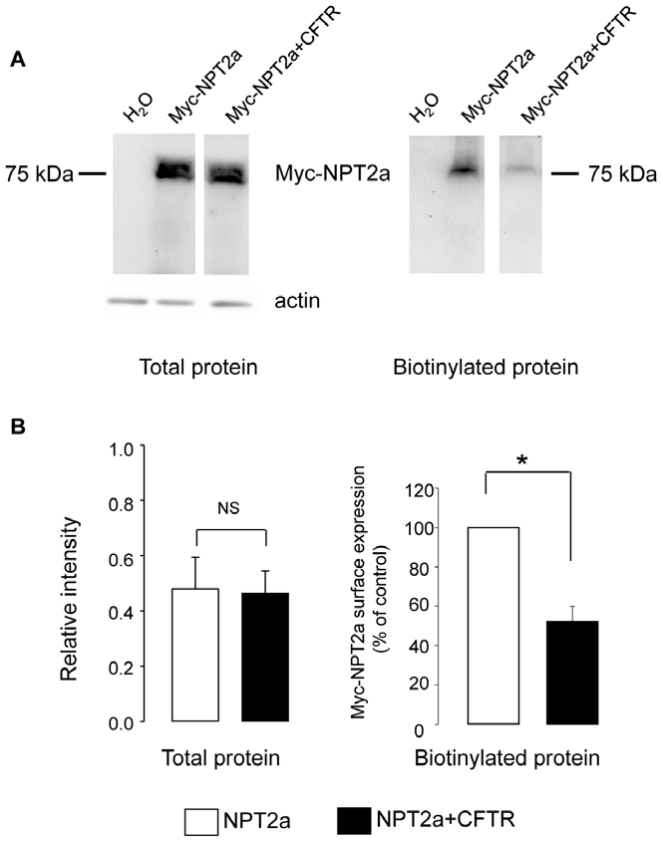
Immunodetection of Myc-NPT2a protein expressed in Myc-NPT2a- and Myc-NPT2a+CFTR-oocytes. A **:** Myc-NPT2a expression in Myc-NPT2a- and Myc-NPT2a+CFTR-oocytes (total or biotinylated proteins) was analyzed by Western blot using a polyclonal Myc antibody. H_2_O-injected oocytes were used as control. Actin expression was used as loading control of total proteins. The molecular weight of Myc-NPT2a is indicated. B: Left part: Quantification of the staining intensity of Myc-NPT2a in total lysate over the staining intensity of actin from Myc-NPT2a-oocytes (white column) and NPT2a+CFTR-oocytes (black column). Results are expressed as means ± SEM (N = 4). Right part: Quantification of the staining intensity of Myc-NPT2a cell surface expression from Myc-NPT2a+CFTR oocytes (black column) normalized against the staining intensity of Myc-NPT2a cell surface expression from Myc-NPT2a-oocytes. Results are expressed as means ± SEM (N = 4); *: P<0.05.

To determine if CFTR expression modified the basic kinetic properties of NPT2a co-transport, these properties were characterized in oocytes expressing NPT2a alone or in combination with CFTR. NPT2a’s apparent K_m_ for its substrates was not affected by CFTR expression (expressed as mean ±SD from N = 5 experiments, K_m_ for Pi was 0.07± 0.02 in NPT2a-oocytes *vs* 0.06 ± 0.02 mM in NPT2a+CFTR-oocytes; K_m_ for Na^+^ was 40.7 ± 14.6 in NPT2a-oocytes *vs* 41.8 ± 13.4 mM in NPT2a+CFTR-oocytes) (**Figure 2A and B**). The pH sensitivity of the transporter was not changed by the co-expression of CFTR (**Figure 2** **inset**). In contrast, CFTR co-expression significantly reduced the IPi_max_ (reflecting the apparent V_max_ of the co-transporter) of NPT2a (**Figure 2**), suggesting a decrease in its membrane expression. These results were supported by immunoblots: **Figure 3A** shows a reduced Myc-NPT2a level in the plasma membranes of oocytes expressing both Myc-NPT2a and CFTR, compared to oocytes expressing Myc-NPT2a alone, whereas the level of Myc-NPT2a protein from total cell lysates was the same in the two samples. Four separate experiments (analyzed in duplicate) showed similar decreases in Myc-NPT2z membrane expression from Myc-NPT2a+CFTR-oocytes, as summarized in **Figure 3B**.

### PKA Stimulation Increases NPT2a-mediated Transport in Oocytes Co-expressing NPT2a and CFTR

**Figure 4:**
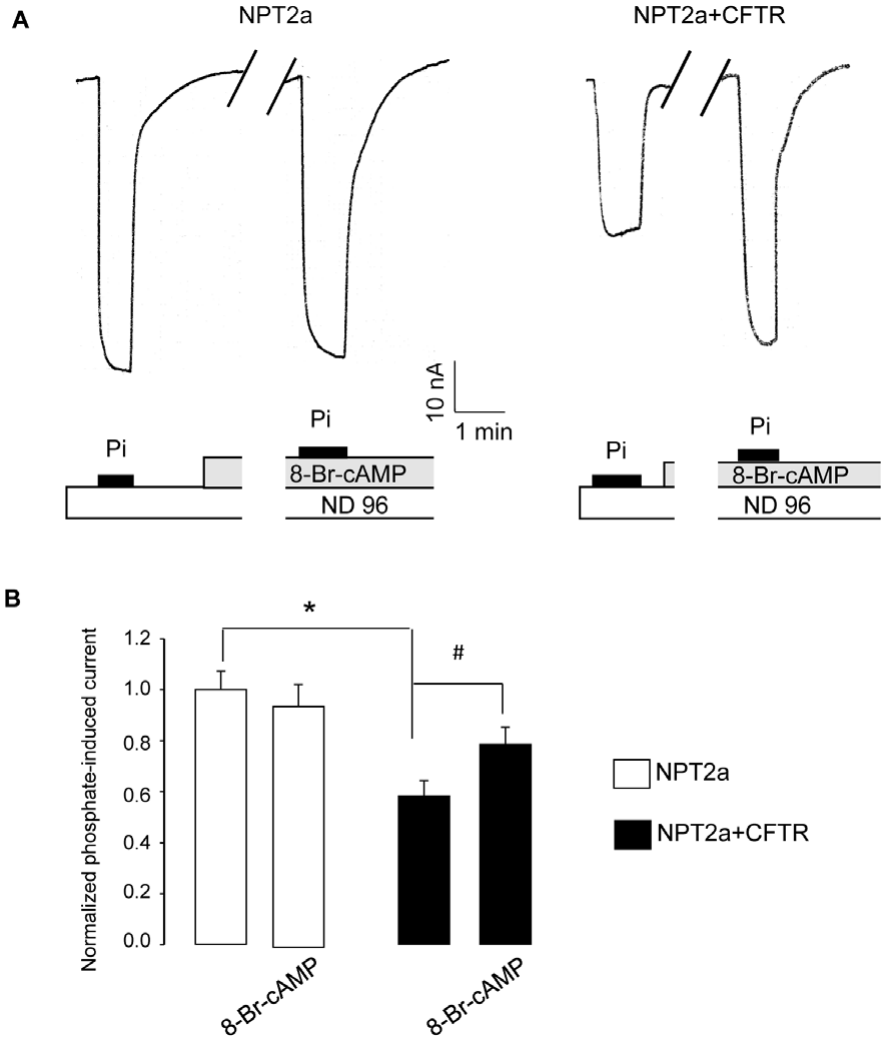
Effect of a permeant cAMP analog on the phosphate-induced current in NPT2a-oocytes or NPT2a+CFTR-oocytes. Phosphate-induced current (IPi) was measured in voltage-clamped (−50 mV) NPT2a- or co-and NPT2a+CFTR-oocytes. A. Original tracings obtained from a NPT2a-oocyte (left), and from a NPT2a+CFTR-oocyte (right), exposed in a reversible manner to 1 mM Pi, as indicated by a black bar. As indicated below the tracings, IPi was first induced in basal condition, ^basal^IPi (superfusion of ND96 supplemented with 1 mM Pi), then in an experimental condition, ^exptl^IPi, herein after 10 min exposure to 8 Bromoadenosine-3′5′-cyclic monophosphate (8-Br-cAMP, 100 µM). The break in each trace represents a ∼ 9 min exposure to the cAMP agonist that is not shown. B. Summary of the results (means ± SEM) of the effect of 8-Br-cAMP, 100 µM, on IPi in NPT2a-oocytes (n  =  8, white columns) and in NPT2a+CFTR-oocytes (n =  8, black columns) from N =  3 experiments. Measured IPi were normalized against ^basal^IPi from NPT2a-oocytes from the same batch of oocytes. The significance of the difference between Pi-induced currents measured in basal or in experimental conditions in NPT2a+CFTR-oocytes and ^basal^IPi from NPT2a-oocytes was analyzed using unpaired Student’s *t*-test (*: P < 0.05). The significance of the difference between ^basal^IPi and ^exptl^IPi within the same type of oocytes was assessed using paired Student’s *t*-test (#: P < 0.05).

After having determined the basic properties of NPT2a in the presence of CFTR, we turned to investigate if CFTR expression may have affected NPT2a regulation. IPi was measured in a paired fashion before and during the stimulation of kinases A and C, in NPT2a- and in NPT2a+CFTR oocytes. Up to 30 min exposure of NPT2a-oocytes to the Forsk+IBMX mixture or to a permeant cAMP had no effect on IPi (−65.1±4.7 in the control condition *vs* −61.6±5.6 nA in the stimulating condition, n =  23) whereas PMA 50 nM gradually decreased IPi (by 46.1±6.9% after 20 min of exposure, n = 9). These results agree with previous studies on the regulation of NPT2a in *X. laevis* oocytes [20], [21], [22]. In NPT2a+CFTR-oocytes, activation of PKA or PKC induced a delayed but robust inward current (at Vc = −50 mV) due to CFTR activation, thus IPi was measured before ICFTR occurred. As in NPT2a-oocytes, IPi gradually decreased significantly in the presence of PMA in NPT2a+CFTR-oocytes (n = 8, not shown). But in contrast to that observed in NPT2a-oocytes, the permeant analog of cAMP 8-Br-cAMP (100 µM) induced within ∼ 10 min of exposure a significant increase in IPi in NPT2a+CFTR-oocytes. **Figure 4** shows original tracings of Pi-induced currents in NPT2a- and NPT2a+CFTR-oocytes before and during 8-Br-cAMP exposure, and summarize results from this experimental series. The synchronous 8-Br-cAMP-induced activation of CFTR channel in NPT2a- and NPT2a+CFTR-oocytes from a same batch is shown in **Figure S2**. Preventing CFTR activation by using Inh*172 (20 µM) did not inhibit the 8-Br-cAMP-induced IPi increase in NPT2a+CFTR-oocytes (**Figure S3)**. Using Forsk+IBMX in place of 8-Br-cAMP also raised IPi : within 5 min of exposure, IPi increased from –28.2±2.3 to –42.7±2.9, n  = 26); this significant increase was prevented in the presence of 50 µM H89 (n = 5). Using non-permeant cAMP (100 µM) was without effect on IPi (n  =  4), ruling out the involvement of a putative cAMP membrane receptor in this response [23].

**Figure 5:**
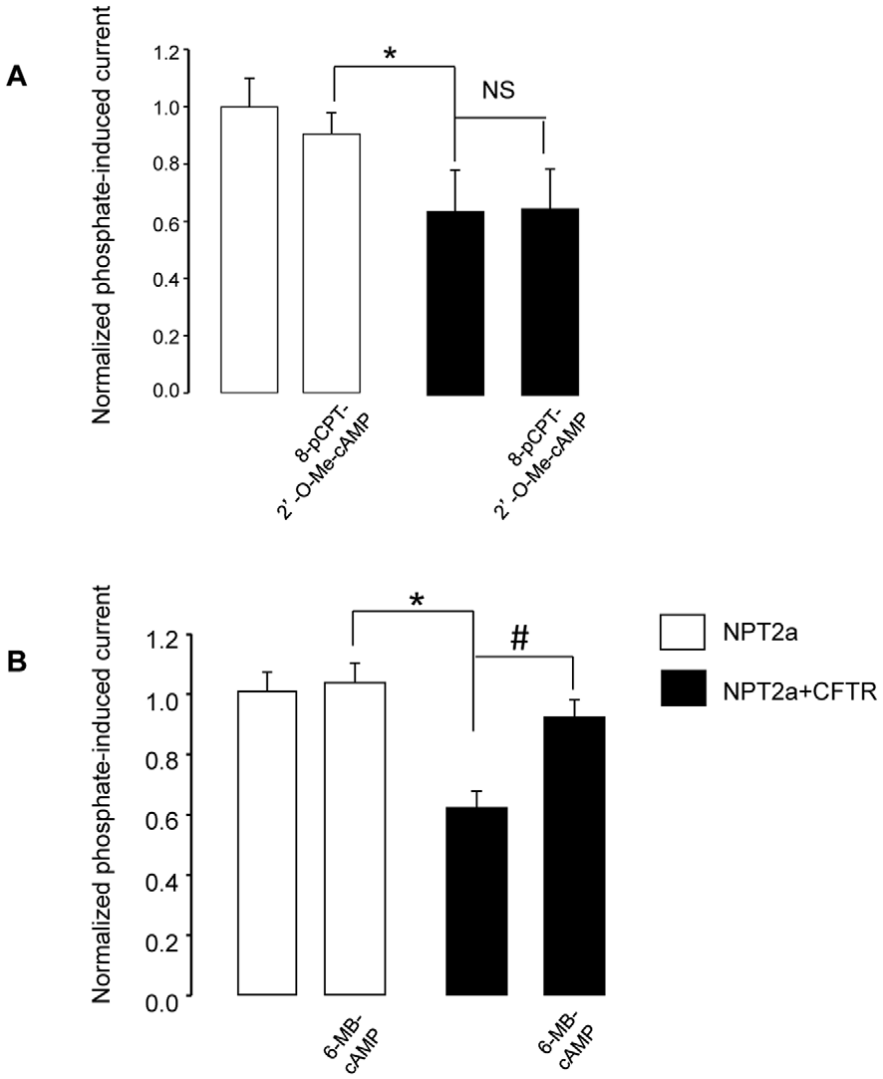
Effects of agonists of EPAC and PKA pathways on NPT2a function and surface expression. Phosphate-induced currents (IPi) were evoked in voltage-clamped (–50 mV) cells by the exposure of NPT2a-oocytes (white columns), or NPT2a+CFTR-oocytes (black columns) to 1 mM Pi. As above, IPi was first induced in basal condition, ^basal^IPi, then after the activation of a cAMP-dependent signaling pathway, ^exptl^IPi, during 10 min (panels A and B). Results are as means ± SEM. IPi were normalized against ^basal^IPi from NPT2a-oocytes from the same batch of oocytes. The significance of the difference between ^basal^IPi from NPT2a-oocytes and ^basal^IPi or ^exptl^IPi from NPT2a+CFTR-oocytes was analyzed using unpaired Student’s *t*-test (*: P < 0.05). The significance of the difference between ^basal^IPi and ^exptl^IPi within the same type of oocytes was assessed using paired Student’s *t*-test (#: P < 0.05). A: Effect of para-Chlorophenylthio-2′-O-methyladenosine-3′, 5′-cyclic monophosphate (8-pCPT-2′-O-Me-cAMP, 25 µM), an activator on the EPAC pathway on IPi in NPT2a- and NPT2a+CFTR-oocytes (n  =  9 for each type; N =  2). B: Effect of N^6^-monobutyryladenosine-3′, 5′-cyclic monophosphate (6-MB-cAMP, 25 µM), an activator of the PKA pathway on IPi in NPT2a- and NPT2a+CFTR-oocytes (n  =  19 and n  =  22, respectively; N =  6).

**Figure 6:**
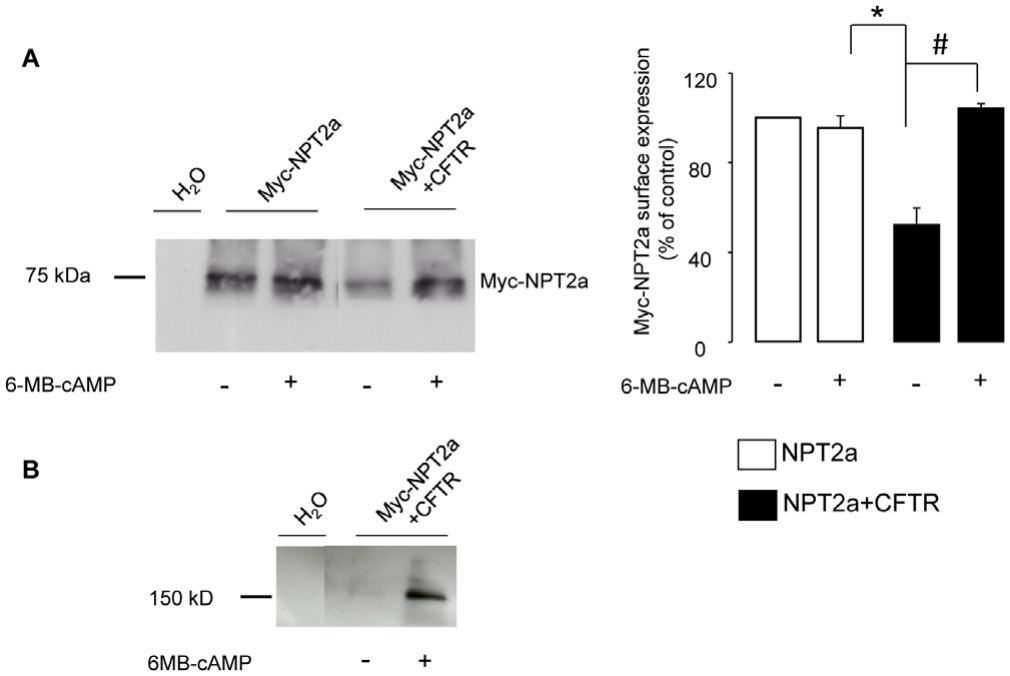
Effect of PKA stimulation on NPT2a and CFTR cell surface expression. Myc-NPT2a- and Myc-NPT2a+CFTR-oocytes from a same batch were kept in control condition or submitted to 25 µM of 6-MB-cAMP for 15 min, as indicated below the panel. Control oocytes were H_2_O-injected. A: Effect of PKA stimulation on NPT2a cell surface expression. Left: Cell surface biotinylated proteins were probed with an anti-Myc antibody; the molecular weight of Myc-NPT2a is indicated on the figure. Right: Results from 4 separate experiments, were quantified. The staining intensity of Myc-NPT2a cell surface expression from Myc-NPT2a+CFTR oocytes (black columns) was normalized against the staining intensity of Myc-NPT2a cell surface expression from Myc-NPT2a-oocytes (white columns) in basal condition. The significance of the difference between the results was assessed using unpaired (*: P < 0.05) or paired (#: P < 0.05) Student’s *t*-test. B: Effect of PKA stimulation on CFTR cell surface expression. Cell surface biotinylated proteins were probed with an anti-CFTR antibody; the molecular weight of CFTR is indicated on the figure. Similar results were obtained in 2 independent experiments.

To better define the signaling mechanism that underlies the cAMPi-induced increase in IPi in NPT2a+CFTR-oocytes, we tested the effect of various membrane permeant analogs of cAMP, using concentrations ≤25 µM to ensure their selectivity as regards to PKA holoenzymes *vs* Exchange Protein directly Activated by cAMP (EPAC) [24], [25]. Exposure of NPT2a+CFTR-oocytes to the EPAC activator 8-pCPT-2′-O-Me-cAMP (25 µM) did not affect IPi (**Figure 5A**). Also, the PKAII activator 6-MBC-cAMP (25 µM) was without effect on IPi in NPT2a- (n  = 9) as well as in NPT2a+CFTR-oocytes exposed to 6-MBC-cAMP (n  =  9) or to the 6-MBC-cAMP-containing solution supplemented with 8-Sp-PIP-cAMP (n = 6). In contrast, exposure to the PKAI activator 6-MB-cAMP (25 µM) significantly increased IPi (by 30.1±5.8%, n  = 22) in NPT2a+CFTR-oocytes, but not in NPT2a-oocytes as shown in **Figure 5B**. Adding 8-HA-cAMP to the 6-MB-cAMP-containing solution increased IPi in NPT2a+CFTR-oocytes by 33.7±3.1% (n = 9), not different from the effect of 6-MB-cAMP alone. A 2-fold lower concentration of 6-MB-cAMP also significantly increased IPi in NPT2a+CFTR oocytes (by 38±2% n = 6). These results suggest that PKA stimulation enhances the activity of NPT2a when CFTR is present. To determine if the increase in NPT2a function (after stimulation of PKA) was related to its enhanced membrane expression, we performed Western blot analysis of proteins from biotinylated plasma membranes of both Myc-NPT2a- and Myc-NPT2a+CFTR-oocytes (stimulated or not). An increased level of NPT2a proteins was detected when NPT2a+CFTR-oocytes had been exposed to 6-MB-cAMP (25 µM, 15 min), compared to control (untreated) NPT2a+CFTR-oocytes from the same batch (**Figure 6A**). Similar results were obtained by using the Forsk+IBMX mixture or 8-Br-cAMP (not shown). These results suggest that, in the presence of CFTR, increasing cAMPi may increase the trafficking of NPT2a to the plasma membrane. A cAMPi-regulated mechanism of CFTR trafficking to the oolemma was previously reported [11] and is in agreement with the robust increase in CFTR plasma membrane level that is observed after stimulating NPT2a+CFTR-oocytes by 6-MB-cAMP (**Figure 6B**). To support the hypothesis of an increased trafficking of NPT2a, we used brefeldin A (BFA) to inhibit the insertion of protein to the membrane of *X. laevis* oocytes [26], [27]. IPi was measured in NPT2a+CFTR- oocytes from the same batch that have been incubated or not in a BFA-containing medium (ND96 supplemented with 18 µM of BFA). The BFA-incubation time was limited to 2–3 hours, i.e., a condition that did not modify basal IPi (preliminary experiments showed that incubation >4 hours decreased IPi, showing that the disequilibrium between protein insertion and retrieval was reached). Under this experimental condition, 8-Br-cAMP (100 µM) no longer stimulated IPi (n  = 14), contrasting with the 26±2.8% increase in IPi measured in NPT2a+CFTR-oocytes that had not been incubated with BFA (n =  13). Taken together, these results are consistent with a cAMPi-induced trafficking of NPT2a- in NPT2a+CFTR-oocytes.

### Further Evidence for a Functional Interaction between NPT2a and CFTR in X. Laevis Oocytes

**Figure 7:**
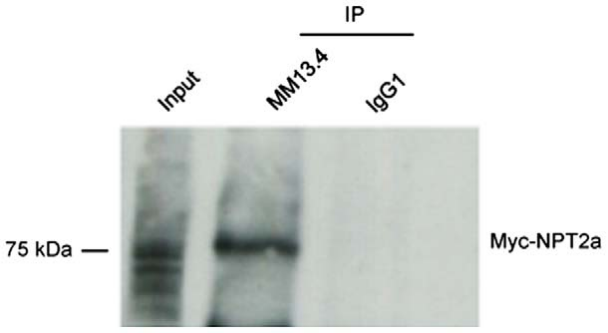
NPT2a and CFTR expressed in *X. laevis* oocytes co-immunoprecipitate. Representative experiment showing the co-immunoprecipitation of NPT2a and CFTR after their expression in *X. laevis* oocytes. Proteins from oocytes co-expressing Myc-NPT2a and CFTR were immunoprecipitated with MM13-4 Ab, or with IgG1 Ab used as a negative control. They were probed with the anti-Myc antibody. The molecular weight of the detected protein is indicated Similar results were obtained in N  =  3 independent experiments.

**Figure 8:**
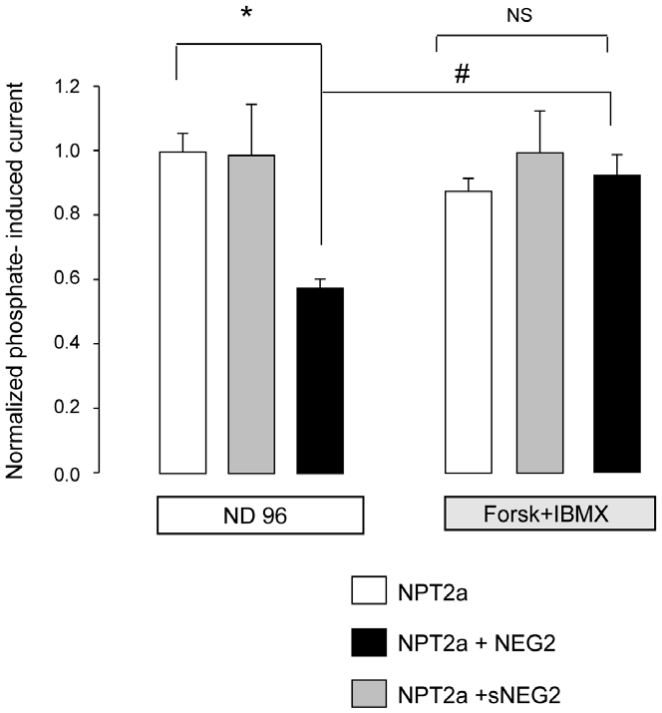
Effect of injecting NEG2 peptide on NPT2a function. The current induced by 1 mM phosphate (IPi) was measured in voltage-clamped (−50 mV) NPT2a-oocytes and was measured in oocytes injected either with the NEG2 peptide (black column, n  =  12), with a scrambled peptide (sNEG2, grey column, n  =  9), or in non-injected oocytes (white column, n  = 10). IPi was measured in basal condition (^basal^IPi, measured in ND96 medium) then under an experimental stimulating condition (^exptl^IPi, measured after a 10 min-incubation in ND96 supplemented with forskolin, 1µM, and isobutylmethylxanthine, 100 µM, Forsk+IBMX). Results are presented as means ± SEM from N  =  4 experiments. IPi were normalized against ^basal^IPi from control (non injected with a peptide) NPT2a-oocytes from the same batch of oocytes. The significance of the difference was analyzed using unpaired Student’s *t*-test (*: P < 0.05) or paired Student’s *t*-test (#: P < 0.05).

From these results, we hypothesized that the cAMPi-induced trafficking of NPT2a was associated with the cAMPi-induced trafficking of CFTR. If so, protein-protein interactions should exist between CFTR and NPT2a when both proteins are expressed in *X. laevis* oocytes. This was supported by the co-immunoprecipitation of Myc-NPT2a and CFTR using Myc and MM13.4 antibodies (**Figure 7**). To test the possible involvement of NEG2, a short sequence from the CFTR regulatory domain, in the effect of CFTR expression on NPT2a function, IPi was measured in NPT2a-oocytes that were injected with NEG2 or with a scrambled peptide, sNEG2 (used as a negative control). As shown in **Figure 8**, the presence of NEG2 mimicked the effect of CFTR co-expression on IPi: basal IPi value was significantly reduced in NPT2a-oocytes supplemented with NEG2, compared to non-injected NPT2a-oocytes (or to NPT2a-oocytes supplemented with sNEG2). Moreover, the Forsk+IBMX mixture (10 min exposure) enhanced IPi in NEG2- injected NPT2a-oocytes, but was without effect in other oocytes (**Figure 8**). Dot blot assays (**Figure S4**) reinforced the observations that NPT2a proteins and NEG2 peptide are interacting, although, as in the case of co-immunoprecipitation assays, this result does not preclude direct/indirect interactions.

## Discussion

In this study, we tested the hypothesis of a functional interaction between CFTR and NPT2a by expressing both proteins in *X. laevis* oocytes. This model is commonly used in a first approach to determine functional interactions in heterogously expressed proteins. For example, it was shown that in *X. laevis* oocytes, CFTR expression activated the low pH-activated Na^+^ currents mediated by the acid-gated Na^+^ channels ASIC1a/2a [28]. Also, it was shown in this model that CFTR expression reduced the ENaC-mediated amiloride-sensitive current and also that CFTR activation further decreased ENaC activity but the precise mechanism underlying CFTR-induced down-regulation of ENaC remains debated [29], [30], [31], [32], [33]. It has been also argued that the CFTR-associated reduction in amiloride-sensitive current in *X. laevis* oocytes was an only *apparent* reduction, related to a drop in the series resistances that may be produced by the electrical TEVC circuit when CFTR is activated [12], [13]. In the present study, we report that the expression of CFTR reduced IPi. This reduction was measured in basal conditions, i.e., in the absence of CFTR activation. This avoids the problem of a sudden change in intracellular Cl^-^ activity or a large change in membrane conductance being responsible for the decrease in IPi [12], [13], [29], [32]. Other pitfalls were eliminated: NPT2a expression in total proteins was not different in NPT2a-and in NPT2a+CFTR-oocytes and the functional expression of NPT2a was not delayed by CFTR co-expression. Thus, one can safely conclude that CFTR expression *per se* was associated with a decreased NPT2a function. The decrease in NPT2a function was very likely related to a decrease in NPT2a membrane expression, as shown by kinetics and Western blot analysis (**Figure 2** and **3**).

Then, we turned to investigate if CFTR might modify NPT2a regulation; in these protocols in which PKA and PKC were stimulated, leading to a delayed CFTR activation, we measured IPi before ICFTR occurred. The delayed CFTR activation is consistent with the expression of CFTR in the oocyte intracellular compartment, followed by its trafficking to the membrane upon kinase stimulation. It was previously shown that most of the CFTR-mediated current is related to cAMP -induced recruitment of CFTR from an intracellular pool [11]. This is also supported by results from our laboratory: in Western blot analysis of biotinylated plasma membranes from Myc-NPT2a+CFTR-oocytes (stimulated or not by 6-MB-cAMP), CFTR protein level was nearly undetectable in the non-stimulated condition but strongly stained in the stimulated condition (**Figure 6**). An increase in membrane expression of Myc-NPT2a was also detected in Myc-NPT2a+CFTR-oocytes, and was associated with the enhancement of Pi transport, as shown in **Figure 5**. The cAMPi-induced activation of IPi was detected only in the presence of CFTR, suggesting that the expression of CFTR in oocytes modifies NPT2a regulation. The inhibition of CFTR-mediated transport by Inh*172 did not cancel the cAMP-induced increase in IPi nor NPT2a increased plasma membrane expression (**Figure S3**). These results indicate that CFTR expression rather than CFTR channel activity interacts with NPT2a function. Other CFTR-associated changes (or unmasking events) in the regulation of transporters have been reported in the literature. It was shown that the cAMPi-induced stimulation of CFTR inhibits ENaC by decreasing its open probability, Po [34], [35], whereas in the absence of CFTR, cAMPi stimulates ENaC (both by reducing channel retrieval from the membrane and by increasing Po, [36]). CFTR enhances the glibenclamide sensitivity of ROMK2 activity [37] and induces a gain in cAMPi sensitivity of SLC26A3, which is cAMP-insensitive in the absence of CFTR [38]. In the present study, the cAMPi-induced increase in NPT2a function was related to an enhanced membrane expression of the transporter (**Figure 6**). This was an unexpected finding, because not only experimental conditions that increase cAMPi are without effect on IPi in oocytes expressing only NPT2a ([20], [21], [22] and the present study) but also they induce the membrane retrieval of the transporter in renal proximal tubular cells [39], [40]). Thus, we have reported here a novel aspect of the regulation of NPT2a. This regulation is conferred by CFTR expression, and appears to be mediated by cAMPi. The use of agonists to discriminate between the 2 cAMP-dependent signaling pathways, PKA and EPAC, is consistent with the involvement of cAMPi/PKA stimulation in IPi increase [24], [25], [41]: IPi was stimulated by the PKA activator 8-Br cAMP but not by the EPAC agonist, 8-pCPT-2′-O-Me-cAMP. More precisely, the use of a more specific agonist suggests that PKA I rather than PKA II holoenzyme is involved in IPi stimulation: 6-MB-cAMP (used as a specific activator of the A site of PKAI) but not 6-MBC-cAMP (used as a specific activator of the A site of PKAII) increased IPi in NPT2a+CFTR-oocytes. Using activators of the B sites of each isozyme to achieve a synergistic activation of A and B sites of PKAI did not modify the above results. The failure of the PKAII agonist to stimulate IPi may reflect the specificity of the PKAI effect on NPT2a regulation, and/or a deficit in a specific A kinase anchoring protein (AKAP) to bind the PKA Regulatory subunit RII [42]: the co-expression of AKAP79 was necessary to observe the stimulatory effect of cAMP on ROMK in *X. laevis* oocytes [43]. One may speculate that in oocytes the limiting factor for the regulation of NPT2a by PKA stimulation are also AKAPs since are they involved in the PTH/PKA signaling complex that lead to down regulation of NPT2a in PTH-target cells [43], [44]. However, we cannot formally exclude that other factors than PKA stimulation participate in the observed stimulation of NPT2a function. Despite using low concentrations of agonists to ensure their selectivity [24], [25], undesirable side effects of these drugs remain possible. Phosphodiesterase (PDE) inhibition by cyclic nucleotide analogs has been documented [45]. PDE inhibition would induce a rise in endogenous cAMP, hampering the interpretation of permeant-cAMP effects. In the present study, it seems rather unlikely that PDE inhibition was involved in IPi increase, since it was not observed using all cyclic nucleotide analogs, but only with PKAI agonists and with the highly PDE-hydrolysable derivative 8-Br cAMP. However, we cannot formally exclude that 6MB-cAMP has other effects than PKAI activation in our experimental model.

Nonetheless, the easiest interpretation of results obtained with cAMP derivatives is that the increase in IPi in NPT2a+CFTR-oocytes was mediated by PKAI. However, the downstream targets of PKAI stimulation remain to be clarified. NPT2a presents several consensus phosphorylation sites; however, results on NPT2a as a phosphoprotein are conflicting [46], [47]. By contrast, it is firmly established that CFTR gating is induced by the phosphorylation of multiple residues in the R domain (amino-acids 590-859) by PKA- stimulation (and to a lower extent, by PKC-stimulation, [48]). The targeting by cAMP/PKA of the R domain also regulates CFTR trafficking from intracellular compartments to the plasma membrane. This trafficking, that was observed in various cell models including *X. laevis* oocytes, is related to a region with a high content of negatively charged amino acids, NEG2, localized near the C terminal of R domain [11], [49], [50], [51], [52]. We hypothesized that in our study, the trafficking mechanisms that are normally involved in the cAMP-induced trafficking of CFTR also induce the trafficking of NPT2a to the plasma membrane, due to the interaction of both proteins in an intracellular compartment. This hypothesis was supported by several experimental findings. First, PKA stimulation failed to stimulate IPi in NPT2a+CFTR-oocytes that have been BFA-incubated. BFA is a fungal metabolite known to inhibit the secretory pathway of synthesized proteins; it was shown to prevent the cAMP-dependent insertion of CFTR-containing vesicles to the plasma membrane of oocytes [27]. Thus, preventing the trafficking of vesicules containing CFTR inhibits the stimulation of NPT2a. Second, the level of plasma membrane expression of Myc-NPT2a was increased in stimulated NPT2a+CFTR- compared to non-stimulated oocytes, indicating that the cAMPi-induced activation of IPi was related to an increase in NPT2a surface membrane expression (see **Figure 6**). Third, CFTR and NPT2a were co-immunoprecipitated (see **Figure** **7**), supporting a direct or indirect interaction between both proteins.

In an attempt to get insights into the mechanism that links CFTR and NPT2a trafficking, we checked the effect of NEG2 on NPT2a function. When NEG2 was injected into NPT2a-oocytes, IPi was reduced and stimulated upon PKA stimulation. However, the mechanism underlying these results is not clear. It was proposed that in non-stimulating conditions, NEG2 stabilizes CFTR in intracellular vesicles by preventing its trafficking to the membrane, whereas in PKA-stimulated conditions, the phosphorylation of the R domain suppresses the stabilizing effect of NEG2, allowing the fusion of CFTR-containing vesicles with the plasma membrane [11]. In our experiments, the stabilizing effect of NEG2 in vesicules may explain the decrease in basal IPi in NPT2a-oocytes supplemented with NEG2, but the cAMPi-induced stimulation of IPi remains unclear. Because a dual effect (activation and inhibition) of NEG2 on CFTR function has been proposed [50], one might speculate that NEG2 has a dual effect on trafficking: the phosphorylation of NEG2 itself would suppress its own stabilizing effect. Such an hypothesis needs to be further investigated. Also, it would be interesting to investigate the possible effect of protein phosphatases on NPT2a: it was recently reported that protein phosphatase 2A, known to dephosphorylate CFTR, modulates the trafficking of the renal Na,K-ATPase pump [53]). Recent studies have explored the signaling and the mechanisms of NPT2a trafficking to the membrane in response to acute low phosphate diet and to insulin/insulin growth factor 1, that increase tubular phosphate reabsorption [54], [55]. Our study sheds light on a novel, CFTR-associated, regulatory mechanism for NPT2a in *X. laevis* oocytes, and raises the question of the role of CFTR in the trafficking of other transport systems. We might speculate by focusing on CFTR and NPT2a interaction that, if a CFTR-associated cAMP-regulated trafficking occurs in native tissue, NPT2a-exocytosis participates in PTH resistance of respiratory alkalosis that is mediated by beta adrenergic receptors signaling [56]. Thus, the consequences of CFTR and NPT2a interactive regulatory processes should be further explored in physiology and during CF.

## Supporting Information

Figure S1 **Current/voltage (I/V) relationships in CFTR-oocytes.** Two-electrode voltage-clamp experiments were performed in oocytes expressing CFTR, CFTR-oocytes. From resting membrane potential, voltage steps of ± 20 mV were applied in the –100 to +60 mV range in various experimental conditions. Results are presented as means±SEM, *: P<0.05. A: Current values at -100/+60 mV were measured in control conditions (ND96 superfusion, white columns), and in stimulated conditions using permeant cAMP analog 8 Bromoadenosine-3′5′-cyclic monophosphate, 8-Br-cAMP, 100 µM (black columns n  =  12 oocytes from N = 3 experiments). In a separate series (n  =  10 oocytes from N =  3 experiments) stimulation was achieved using forskolin, 1 µM (Forsk) plus isobutylmethylxanthine (IBMX), 100 µM (ND96 white columns, Forsk+ IBMX, grey columns). Results were analyzed using paired Student’s t test. *: P<0.05. B: I/V curves from CFTR-oocytes. Oocytes (n  =  12 from N =  3 experiments) were superfused with ND96 (empty circles), then with 100 µM 8-Br-cAMP (full circles). Finally, 20 µM of the CFTR inhibitor CFTR-Inh*172 was added (triangles).
(TIF)

Figure S2 **Time course of CFTR activation in CFTR- and NPT2a+CFTR-oocytes.** Original tracings obtained from a CFTR-oocyte (upper panel) and from a NPT2a+CFTR-oocyte (lower panel) from the same batch, showing that CFTR current is activated within the same delay in both types of oocytes. Voltage-clamped oocytes (Vc =  −50 mV) were exposed in a reversible manner to 1 mM Pi (indicated by black bars below the tracings) before and after a 7–8 min exposure (represented by the break) to 8 Bromoadenosine-3′5′-cyclic monophosphate (8-Br-cAMP, 100 µM, indicated by the grey bar below the tracings). Pi exposure had no effect in the CFTR-oocyte. In the NPT2a+CFTR oocyte, Pi exposure induced an inward current that increased upon exposure to the cAMP analog. Further exposure to 8-Br-cAMP induced the activation of CFTR channel, as shown by the slowly occurring inward current, ICFTR. The recording was stopped before the full amplitude of this current was reached: when necessary, ICFTR was assessed by switching from – 50 mV continuous voltage-clamp to current/voltage analysis (see Figure S1).
(TIF)

Figure S3 **Effect of a permeant cAMP analog on NPT2a function and expression in the presence of a CFTR inhibitor.** A: Effect of 8-Br-cAMP plus Inh*172 on phosphate-induced currents (IPi). IPi was measured in voltage-clamped (−50 mV) condition in NPT2a-oocytes (n  =  8, white columns) and in NPT2a+CFTR-oocytes NPT2a+CFTR-oocytes (n =  9, black columns). Results from N =  2 experiments are shown. IPi was measured in the presence of the CFTR inhibitor Inh*172 (20 µM) before and after exposure to 8 Bromoadenosine-3′5′-cyclic monophosphate, (8-Br-cAMP, 100 µM). Significance of the difference was analyzed using unpaired (*: P < 0.05), or paired (#: P < 0.05) Student’s t-test. B: Effect of 8-Br-cAMP plus Inh*172 on NPT2a cell surface expression. Cell surface biotinylated proteins from Myc-NPT2a- and Myc-NPT2a+CFTR-oocytes were probed with an anti-Myc antibody; the molecular weight of Myc-NPT2a is indicated on the figure. Control oocytes were injected with H_2_O.
(TIF)

Figure S4 **Dot blot assays support the binding of NPT2a to NEG2.** The NEG2 peptide, or a scrambled peptide (both dissolved in intracellular-like medium) was spotted on a nitrocellulose membrane and immobilized by drying. Non-specific binding was prevented by applying 1% BSA and 1% nonfat milk in PBST (1 h, RT). Dots were incubated for 1h (RT) in the presence of total proteins extracted from 30 Myc-NPT2a-oocytes, or control (H_2_O-) oocytes. After careful washing with PBST (3 times, 10 min each), anti-Myc Ab (diluted to 1/5000) was applied for 1 h (RT). Detection was carried out using an anti-mouse (diluted to 1/5000) secondary Ab coupled to HRP. Each experiment was performed in triplicate. Similar results were obtained in 3 other separate experiments, each experiment being performed in triplicate.
(TIF)

## References

[1] Wang Y, Soyombo AA, Shcheynikov N, Zeng W, Dorwart M (2006). Slc26a6 regulates CFTR activity in vivo to determine pancreatic duct HCO_3_
^-^ secretion: relevance to cystic fibrosis.. EMBO J.

[2] Song Y, Namkung W, Nielson DW, Lee JW, Finkbeiner WE (2009). Airway surface liquid depth measured in ex vivo fragments of pig and human trachea:dependence on Na^+^ and Cl^-^ channel function.. Am J Physiol Lung Cell Mol Physiol.

[3] Devuyst O, Guggino WB (2002). Chloride channels in the kidney: lessons learned from knockout animals.. Am J Physiol Renal Physiol.

[4] Jouret F, Bernard A, Hermans C, Dom G, Terryn S (2007). Cystic fibrosis is associated with a defect in apical receptor-mediated endocytosis in mouse and human kidney.. J Am Soc Nephrol.

[5] Bronckers A, Kalogeraki L, Jorna HJ, Wilke M, Bervoets TJ (2010). The cystic fibrosis transmembrane conductance regulator (CFTR) is expressed in maturation stage ameloblasts, odontoblasts and bone cells.. Bone.

[6] Shead EF, Haworth CS, Condliffe AM, McKeon DJ, Scott MA (2007). Cystic fibrosis transmembrane conductance regulator (CFTR) is expressed in human bone.. Thorax.

[7] Street ME, Spaggiari C, Ziveri MA, Volta C, Federico G (2006). Analysis of bone mineral density and turnover in patients with cystic fibrosis: associations between the IGF system and inflammatory cytokines.. Horm Res.

[8] Katz SM, Krueger LJ, Falkner B (1988). Microscopic nephrocalcinosis in cystic fibrosis.. N Engl J Med.

[9] Gibney EM, Goldfarb DS (2003). The association of nephrolithiasis with cystic fibrosis.. Am J Kidney Dis.

[10] Prié D, Huart V, Bakouh N, Planelles G, Dellis O (2002). Nephrolithiasis and osteoporosis associated with hypophosphatemia caused by mutations in the type 2a sodium-phosphate cotransporter.. N Engl J Med.

[11] Lewarchik CM, Peters KW, Qi J, Frizzell RA (2008). Regulation of CFTR trafficking by its R domain.. J Biol Chem.

[12] Nagel G, Szellas T, Riordan JR, Friedrich T, Hartung K (2001). Non-specific activation of the epithelial sodium channel by the CFTR chloride channel.. EMBO Rep.

[13] Nagel G, Barbry P, Chabot H, Brochiero E, Hartung K (2005). CFTR fails to inhibit the epithelial sodium channel ENaC expressed in *Xenopus laevis* oocytes.. J Physiol.

[14] Taddei A, Folli C, Zegarra-Moran O, Fanen P, Verkman AS (2004). Altered channel gating mechanism for CFTR inhibition by a high-affinity thiazolidinone blocker.. FEBS Lett.

[15] Nijholt I, Blank T, Liu A, Kugler H, Spiess J (2000). Modulation of hypothalamic NMDA receptor function by cyclic AMP-dependent protein kinase and phosphatases.. J Neurochem.

[16] Schwake M, Pusch M, Kharkovets T, Jentsch TJ (2000). Surface expression and single channel properties of KCNQ2/KCNQ3, M-type K^+^ channels involved in epilepsy.. J Biol Chem.

[17] Harris M, Firsov D, Vuagniaux G, Stutts MJ, Rossier BC (2007). A novel neutrophil elastase inhibitor prevents elastase activation and surface cleavage of theepithelial sodium channel expressed in *Xenopus laevis* oocytes.. J Biol Chem.

[18] Simard CF, Bergeron MJ, Frenette-Cotton R, Carpentier GA, Pelchat ME (2007). Homooligomeric and heterooligomeric associations between K^+^-Cl^-^ cotransporter isoforms and between K^+^-Cl^-^ and Na^+^-K^+^-Cl^-^ cotransporters.. J Biol Chem.

[19] Michlig S, Harris M, Loffing J, Rossier BC, Firsov D (2005). Progesterone down-regulates the open probability of the amiloride-sensitive epithelial sodium channel via a Nedd4–2-dependent mechanism.. J Biol Chem.

[20] Hayes G, Busch AE, Lang F, Biber J, Murer H (1995). Protein kinase C consensus sites and the regulation of renal Na/Pi-cotransport expressed in *Xenopus laevis* oocytes.. Pflugers Arch.

[21] Wagner CA, Raber G, Waldegger S, Osswald H, Biber J (1996). Regulation of the human brush border Na^+^/Phosphate cotransporter expressed in *Xenopus* oocytes by intracellular calcium and protein kinase C. Cell Physiol Biochem.

[22] Forster IC, Traebert M, Jankowski M, Stange G, Biber J (1999). Protein kinase C activators induce membrane retrieval of type II Na+-phosphate cotransporters expressed in *Xenopus* oocytes.. J Physiol.

[23] Bankir L, Ahloulay M, Devreotes PN, Parent CA (2002). Extracellular cAMP inhibits proximal reabsorption: are plasma membrane cAMP receptors involved?. Am J Physiol Renal Physiol.

[24] Holz GG, Kang G, Harbeck M, Roe MW, Chepurny OG (2006). Cell physiology of cAMP sensor Epac.. J Physiol.

[25] Honneger KJ, Capuano P, Winter C, Basic D, Stange G (2006). Regulation of sodium-proton excahnger isoform 3 (NHE3) by PKA and exchange protein directly activated by cAMP (EPAC).. Proc Natl Acad Sc U S A.

[26] Shimkets RA, Lifton RP, Canessa CM (1997). The activity of the epithelial sodium channel is regulated by clathrin-mediated endocytosis.. J Biol Chem.

[27] Weber WM, Segal A, Simaels J, Vankeerberghen A, Cassiman JJ (2001). Functional integrity of the vesicle transporting machinery is required for complete activation of CFTR expressed in *Xenopus laevis* oocytes.. Pflugers Arch.

[28] Ji H-L, Jovov B, Bishop L, Mebane HC, Fuller CM (2002). Up-regulated acid-gated Na^+^ channels (ASIC) by cystic fibrosis conductance regulator co-expression in *Xenopus* oocytes.. J Biol Chem.

[29] König J, Schreiber R, Voelcker T, Mall M, Kunzelmann K (2001). The cystic fibrosis transmembrane conductance regulator (CFTR) inhibits ENaC through an increase in the intracellular Cl^-^ concentration.. EMBO Rep.

[30] Ji HL, Chalfant ML, Jovov B, Lockhart JP, Parker SB (2000). The cytosolic termini of the beta- and gamma-ENaC subunits are involved in the functional interactions between cystic fibrosis transmembrane conductance regulator and epithelial sodium channel.. J Biol Chem.

[31] Suaud L, Li J, Jiang Q, Rubenstein RC, Kleyman TR (2002). Genistein restores functional interactions between Delta F508-CFTR and ENaC in *Xenopus* oocytes.. J Biol Chem.

[32] Bachhuber T, König J, Voelcker T, Mürle B, Schreiber R (2005). Cl^-^ interference with the epithelial Na+ channel ENaC.. J Biol Chem.

[33] Suaud L, Yan W, Carattino MD, Robay A, Kleyman TR (2007). Regulatory interactions of N1303K-CFTR and ENaC in *Xenopus* oocytes: evidence that chloride transport is not necessary for inhibition of ENaC.. Am J Physiol Cell Physiol.

[34] Konstas AA, Koch JP, Korbmacher C (2003). cAMP-dependent activation of CFTR inhibits the epithelial sodium channel (ENaC) without affecting its surface expression.. Pflugers Arch.

[35] Yan W, Samaha FF, Ramkumar M, Kleyman TR, Rubenstein RC (2004). Cystic fibrosis transmembrane conductance regulator differentially regulates human and mouse epithelial sodium channels in Xenopus oocytes.. J Biol Chem.

[36] Yang LM, Rinke R, Korbmacher C (2006). Stimulation of the epithelial sodium channel (ENaC) by cAMP involves putative ERK phosphorylation sites in the C termini of the channel’s beta- and gamma-subunit.. J Biol Chem.

[37] McNicholas CM, Guggino WB, Schwiebert EM, Hebert SC, Giebisch G (1996). Sensitivity of a renal K^+^ channel (ROMK2) to the inhibitory sulfonylurea compound glibenclamide is enhanced by coexpression with the ATP-binding cassette transporter cystic fibrosis transmembrane regulator.. Proc Natl Acad Sci U S A.

[38] Chernova MN, Jiang L, Shmukler BE, Schweinfest CW, Blanco P (2003). Acute regulation of the SLC26A3 congenital chloride diarrhoea anion exchanger (DRA) expressed in *Xenopus* oocytes.. J Physiol.

[39] Murer H, Hernando N, Forster I, Biber J (2003). Regulation of Na/Pi transporter in the proximal tubule..

[40] Prié D, Friedlander G (2010). Genetic disorders of renal phosphate transport.. N Engl J Med.

[41] Cunningham R, Biswas R, Brazie M, Steplock D, Shenolikar S (2009). Signaling pathways utilized by PTH and dopamine to inhibit phosphate transport in mouse renal proximal tubule cells.. Am J Physiol Renal Physiol.

[42] Lim CJ, Yousefi N, Amieux PS, McKnight GS, Taylor SS (2007). Alpha4 integrins are type I cAMP-dependent protein kinase anchoring proteins.. Nat Cell Biol.

[43] Ali S, Chen X, Lu M, Xu JZ, Lerea KM (1998). The A kinase anchoring protein is required for mediating the effect of protein kinase A on ROMK1 channels.. Proc Natl Acad Sci U S A.

[44] Khundmiri SJ, Rane MJ, Lederer ED (2003). Parathyroid hormone regulation of type II sodium-phosphate cotransporters is dependent on an A kinase anchoring protein.. J Biol Chem.

[45] Poppe H, Rybalkin SD, Rehmann H, Hinds TR, Tang X-B (2008). Cyclic nucleotide analogs as probes of signaling pathways.. Nat Methods.

[46] Jankowski M, Hilfiker H, Biber J, Murer H (2001). The opossum kidney cell type IIa Na/P(i) cotransporter is a phosphoprotein..

[47] Déliot N, Hernando N, Horst-Liu Z, Gisler SM, Capuano P (2005). Parathyroid hormone treatment induces dissociation of type IIa Na^+^-P(i) cotransporter-Na^+^/H^+^ exchanger regulatory factor-1 complexes.. Am J Physiol Cell Physiol.

[48] Chappe V, Hinkson DA, Zhu T, Chang X-B, Riordan JR (2003). Phosphorylation of protein kinase C sites in NBD1 and the R domain control CFTR channel activation by PKA.. J Physiol.

[49] Ma J (2000). Stimulatory and inhibitory functions of the R Domain on CFTR chloride channel.. News Physiol Sci.

[50] Xie J, Adams LM, Zhao J, Gerken TA, Davis PB (2002). A short segment of the R domain of cystic fibrosis transmembrane conductance regulator contains channel stimulatory and inhibitory activities that are separable by sequence modification.. J Biol Chem.

[51] Howard M, Jilling T, DuVall M (1996). cAMP-regulated trafficking of epitope-tagged CFTR.. Kidney Int.

[52] Bertrand CA, Frizzell RA (2003). The role of regulated CFTR trafficking in epithelial secretion.. Am J Physiol Cell Physiol.

[53] Kimura T, Han W, Pagel P, Nairn AC, Caplan MJ (2011). Protein phosphatase 2A interacts with the Na^+^,K^+^-ATPase and modulates its trafficking by inhibition of its interaction with arrestin.. PlosOne.

[54] Ahmad A, Khundmiri SJ, Pribble F, Merchant ML, Ameen M (2011). Role of the vacuolar ATPase in the trafficking of renal type IIa sodium phosphate cotransporter.. Cell Physiol Biochem.

[55] Föller M, Kempe DS, Boini KM, Pathare G, Siraskar B (2011). PKB/SGK-resistant SGK3 enhances phosphaturia and calciuria.. J Am Soc Nephrol.

[56] Hoppe A, Rybczynska A, Knox FG, Angielski S (1988). Beta-receptors in resistance to phosphaturic effect of PTH in respiratory alkalosis.. Am J Physiol.

