# Supplementary figures and images for: Functional Interaction between CFTR and the Sodium-Phosphate Co-Transport Type 2a in Xenopus laevis Oocytes
Source: PLoS One. 2012 Apr 13;7(4):e34879. doi: 10.1371/journal.pone.0034879 (PMC3325942; doi:10.1371/journal.pone.0034879)

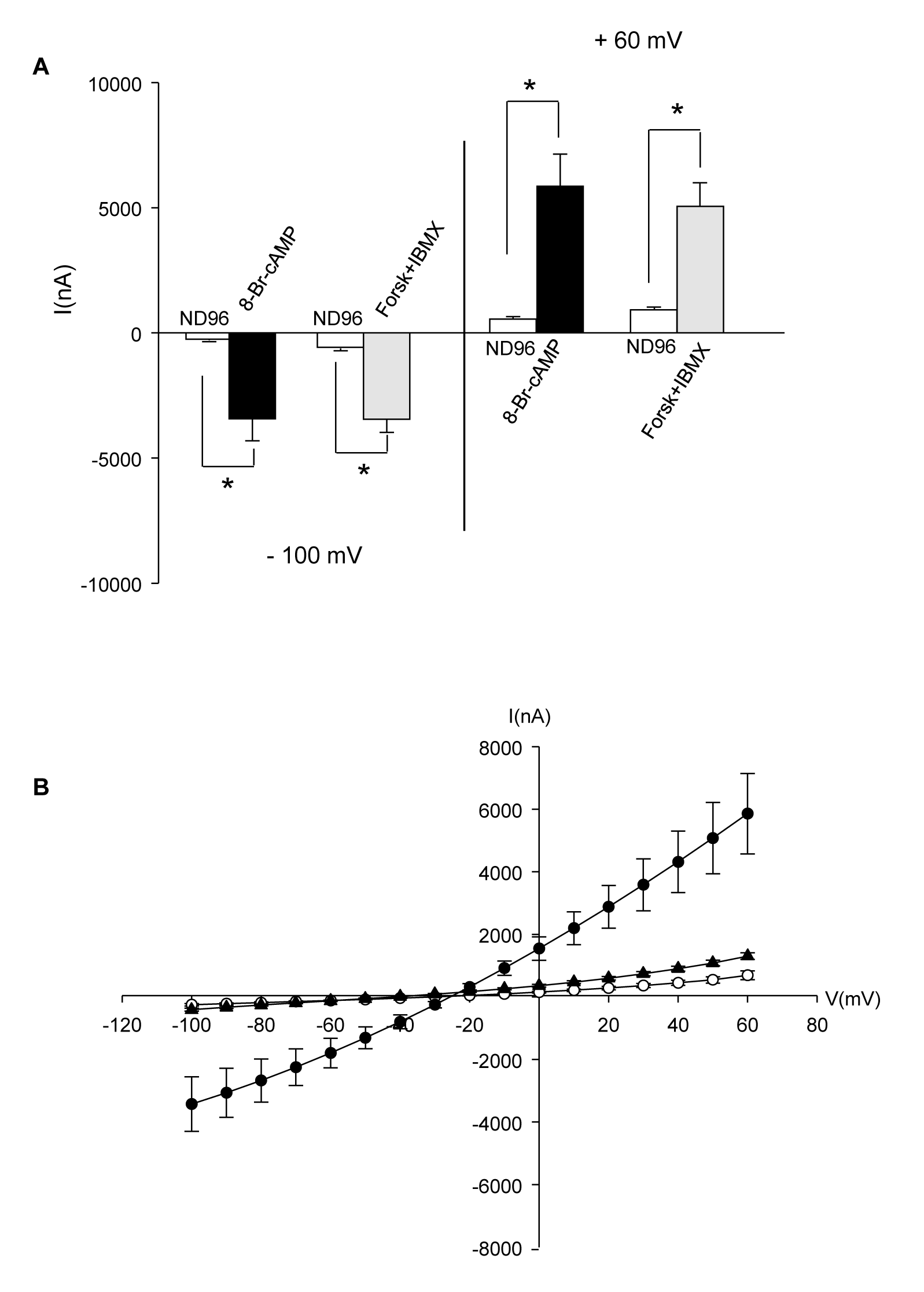

Supplement: Figure S1 — Current/voltage (I/V) relationships in CFTR-oocytes. Two-electrode voltage-clamp experiments were performed in oocytes expressing CFTR, CFTR-oocytes. From resting membrane potential, voltage steps of ± 20 mV were applied in the –100 to +60 mV range in various experimental conditions. Results are presented as means±SEM, *: P<0.05. A: Current values at -100/+60 mV were measured in control conditions (ND96 superfusion, white columns), and in stimulated conditions using permeant cAMP analog 8 Bromoadenosine-3′5′-cyclic monophosphate, 8-Br-cAMP, 100 µM (black columns n = 12 oocytes from N = 3 experiments). In a separate series (n = 10 oocytes from N = 3 experiments) stimulation was achieved using forskolin, 1 µM (Forsk) plus isobutylmethylxanthine (IBMX), 100 µM (ND96 white columns, Forsk+ IBMX, grey columns). Results were analyzed using paired Student’s t test. *: P<0.05. B: I/V curves from CFTR-oocytes. Oocytes (n = 12 from N = 3 experiments) were superfused with ND96 (empty circles), then with 100 µM 8-Br-cAMP (full circles). Finally, 20 µM of the CFTR inhibitor CFTR-Inh*172 was added (triangles). (TIF) [file pone.0034879.s001.tif]

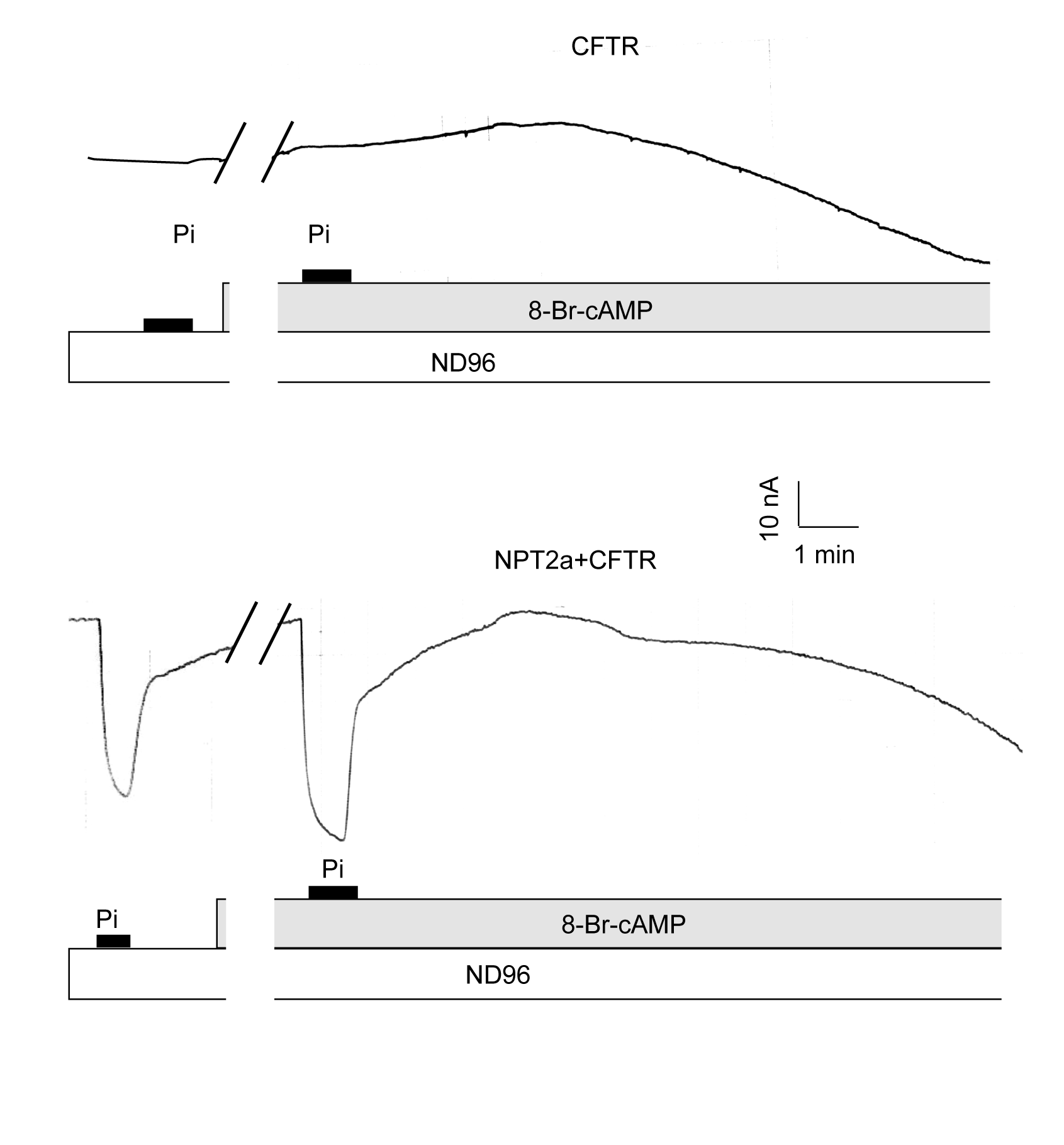

Supplement: Figure S2 — Time course of CFTR activation in CFTR- and NPT2a+CFTR-oocytes. Original tracings obtained from a CFTR-oocyte (upper panel) and from a NPT2a+CFTR-oocyte (lower panel) from the same batch, showing that CFTR current is activated within the same delay in both types of oocytes. Voltage-clamped oocytes (Vc = −50 mV) were exposed in a reversible manner to 1 mM Pi (indicated by black bars below the tracings) before and after a 7–8 min exposure (represented by the break) to 8 Bromoadenosine-3′5′-cyclic monophosphate (8-Br-cAMP, 100 µM, indicated by the grey bar below the tracings). Pi exposure had no effect in the CFTR-oocyte. In the NPT2a+CFTR oocyte, Pi exposure induced an inward current that increased upon exposure to the cAMP analog. Further exposure to 8-Br-cAMP induced the activation of CFTR channel, as shown by the slowly occurring inward current, ICFTR. The recording was stopped before the full amplitude of this current was reached: when necessary, ICFTR was assessed by switching from – 50 mV continuous voltage-clamp to current/voltage analysis (see Figure S1). (TIF) [file pone.0034879.s002.tif]

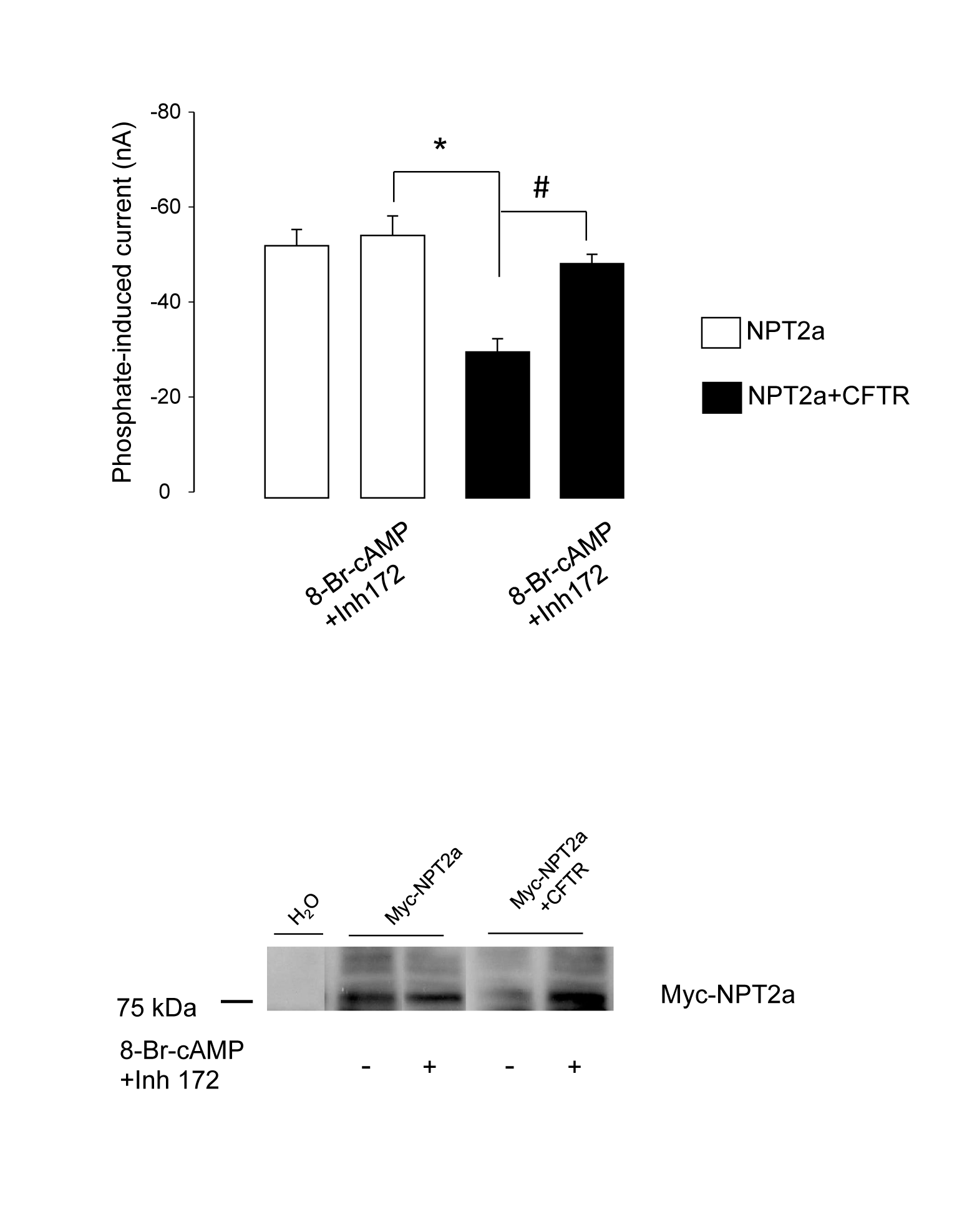

Supplement: Figure S3 — Effect of a permeant cAMP analog on NPT2a function and expression in the presence of a CFTR inhibitor. A: Effect of 8-Br-cAMP plus Inh*172 on phosphate-induced currents (IPi). IPi was measured in voltage-clamped (−50 mV) condition in NPT2a-oocytes (n = 8, white columns) and in NPT2a+CFTR-oocytes NPT2a+CFTR-oocytes (n = 9, black columns). Results from N = 2 experiments are shown. IPi was measured in the presence of the CFTR inhibitor Inh*172 (20 µM) before and after exposure to 8 Bromoadenosine-3′5′-cyclic monophosphate, (8-Br-cAMP, 100 µM). Significance of the difference was analyzed using unpaired (*: P < 0.05), or paired (#: P < 0.05) Student’s t-test. B: Effect of 8-Br-cAMP plus Inh*172 on NPT2a cell surface expression. Cell surface biotinylated proteins from Myc-NPT2a- and Myc-NPT2a+CFTR-oocytes were probed with an anti-Myc antibody; the molecular weight of Myc-NPT2a is indicated on the figure. Control oocytes were injected with H2O. (TIF) [file pone.0034879.s003.tif]

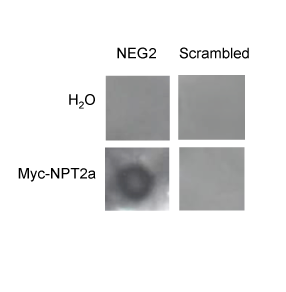

Supplement: Figure S4 — Dot blot assays support the binding of NPT2a to NEG2. The NEG2 peptide, or a scrambled peptide (both dissolved in intracellular-like medium) was spotted on a nitrocellulose membrane and immobilized by drying. Non-specific binding was prevented by applying 1% BSA and 1% nonfat milk in PBST (1 h, RT). Dots were incubated for 1h (RT) in the presence of total proteins extracted from 30 Myc-NPT2a-oocytes, or control (H2O-) oocytes. After careful washing with PBST (3 times, 10 min each), anti-Myc Ab (diluted to 1/5000) was applied for 1 h (RT). Detection was carried out using an anti-mouse (diluted to 1/5000) secondary Ab coupled to HRP. Each experiment was performed in triplicate. Similar results were obtained in 3 other separate experiments, each experiment being performed in triplicate. (TIF) [file pone.0034879.s004.tif]
